# Reconstruction and analysis of potential biomarkers for hypertrophic cardiomyopathy based on a competing endogenous RNA network

**DOI:** 10.1186/s12872-022-02862-9

**Published:** 2022-09-22

**Authors:** Jin-yan Chen, Zhang-xin Xie, Jia-zhen Dai, Jun-yong Han, Kun Wang, Li-hong Lu, Jing-jun Jin, Shi-jie Xue

**Affiliations:** 1 Institute for Immunology, Fujian Academy of Medical Sciences, No. 7 Wusi Road, Fuzhou, 350001 China; 2 Shengli Clinical Medical College of Fujian Medical University, Fuzhou, 350001 China; 3 Department of Emergency, Fujian Provincial Hospital, Fuzhou, 350001 China; 4 Fujian Provincial Key Laboratory of Medical Analysis, Fuzhou, 350001 China; 5 Department of Cardiology, Zhangzhou Affilated Hospital, Zhangzhou, China

**Keywords:** Hypertrophic cardiomyopathy, LINC00310, Competitive endogenous RNAs, Microarray, Sequencing

## Abstract

Hypertrophic cardiomyopathy (HCM) is a common heritable cardiomyopath. Although considerable effort has been made to understand the pathogenesis of HCM, the mechanism of how long noncoding RNA (lncRNA)-associated competing endogenous RNA (ceRNA) network result in HCM remains unknown. In this study, we acquired a total of 520 different expression profiles of lncRNAs (DElncRNAs) and 371 messenger RNAs (mRNA, DEGs) by microarray and 33 microRNAs (DEmiRNAs) by sequencing in plasma of patients with HCM and healthy controls. Then lncRNA–miRNA pairs were predicted using miRcode and starBase and crossed with DEmiRNAs. MiRNA–mRNA pairs were retrieved from miRanda and TargetScan and crossed with DEGs. Combined with these pairs, the ceRNA network with eight lncRNAs, three miRNAs, and 22 mRNAs was constructed. lncRNA RP11-66N24.4 and LINC00310 were among the top 10% nodes. The hub nodes were analyzed to reconstruct a subnetwork. Furthermore, quantitative real-time polymerase chain reaction results showed that LINC00310 was significantly decreased in patients with HCM. For LINC00310, GO analysis revealed that biological processes were enriched in cardiovascular system development, sprouting angiogenesis, circulatory system development, and pathway analysis in the cGMP-PKG signaling pathway. These results indicate that the novel lncRNA-related ceRNA network in HCM and LINC00310 may play a role in the mechanism of HCM pathogenesis, which could provide insight into the pathogenesis of HCM.

**Supplementary Information:**

The online version contains supplementary material available at 10.1186/s12872-022-02862-9.

## Introduction

Hypertrophic cardiomyopathy (HCM) is a common heritable cardiomyopath caused by mutations in genes encoding sarcomeric proteins. The characteristics of HCM includes increased left ventricular (LV) wall thickness to alleviate wall stress and keep normal cardiac function caused by pathological pressure overload [1]. Most of methodologically diverse studies have shown a prevalence of unexplained increase in LV thickness in the range of 0.02–0.23% in adults in North America, Europe, Asia, and Africa, and the prevalence of HCM in different racial groups is similar [2]. HCM and its disastrouscomplications are the common cause of sudden cardiac death in young adults, including athletic individuals [3]. Although genetic discoveries can promote the elucidation of the molecular basis of HCM and a growing number of molecular regulators in HCM have been studied, the underlying mechanism remains unclear. Therefore, further exploration of the complex interaction between genetic and nongenetic factors in HCM is important, such as transcriptional or epigenetic factors.

Long non-coding RNAs (lncRNAs) are defined as RNAs longer than 200 nucleotides, which are not translated into functional proteins. Statistics from NONCODEv6 (http://www.noncode.org/analysis.php) show a total of 173,112 human lncRNA transcripts, including lncRNAs transcribed by RNA polymerase II (Pol II) and other RNA polymerases, lncRNAs from intergenic regions (lincRNAs), and sense or antisense transcripts that overlap with other genes [4]. With the discovery of several lncRNAs, the functions of lncRNAs in multiple disease are well documented. Given their versatility, several lncRNAs affect the transcription of nearby genes and certain chromatin biology, such as DNA damage and repair or DNA replication. Other lncRNAs are structural and/or regulatory, which participate in every process of mRNA life, including splicing, signaling pathways, turnover, and translation [4]. Consequently, lncRNAs affect numerous cellular physiological processes, and alteration of their expression leads to diseases. Thus, several lncRNAs may be used as potential disease biomarkers or therapeutic targets.

A growing body of evidence suggests that lncRNAs could repress target mRNAs through miRNA response elements of the lncRNA-associated competing endogenous RNA (ceRNA) network and serve as a crucial regulatory mechanism in many diseases [5]. Studies have shown several lncRNA-related ceRNA networks in HCM, such as cardiac hypertrophy-related factor (CHRF) [6], ROR [7], H19 [8], Plscr4 [9], myocardial infarction-associated transcript [10], maternally expressed gene three (MEG3) [11], and cytoskeleton regulator RNA (CYTOR) [12]. However, the expression profile of lncRNAs and lncRNA-related ceRNA network in the progression of HCM has not been recognized and thoroughly characterized.

Therefore, we conducted a microarray profile of lncRNAs, mRNAs, and miRNAs in plasma samples from patients with HCM compared with those in healthy control samples. Furthermore, differentially expressed lncRNAs and the lncRNA-miRNA-mRNA network were analyzed on the basis of the ceRNA theory. These results can be used to understand the role of lncRNA in the molecular mechanism and potential therapeutic target for HCM.

## Materials and methods

### Patients

This study was approved by the Ethics Committee of Fujian Provincial Hospital, and all subjects provided written informed consent following the Declaration of Helsinki. The clinical diagnostic criteria of HCM were based on the 2014 European Society of Cardiology Guidelines: HCM in adults was defined by a wall thickness of ≥ 15 mm in one or more LV myocardial segments as measured by any imaging technique (echocardiography, cardiac magnetic resonance imaging, or computed tomography), which is not explained solely by loading conditions [3]. Controls consisted of healthy subjects matched by sex and age without cardiac diseases. The exclusion criteria were as follows: history of hypertension for over 10 years, rheumatic disease, aortic stenosis, congenital heart and metabolic diseases (such as myocardial amyloidosis, Danon disease, and Pompe disease), cardiac hypertrophy of athletes, other organic heart diseases, trauma within 6 months, diabetes, surgery, cancer, or renal dysfunction.

### Plasma collection and RNA isolation

The venous blood of patients with HCM and healthy controls was collected into BD Vacutainer venous blood collection tubes containing ethylenediaminetetraacetic acid. The plasma was separated, and total RNA from plasma was isolated using TRIzol LS Reagent (Invitrogen, Carlsbad, CA, USA) according to the manufacturer’s instructions. The quantity and integrity of RNA were measured using a NanoDrop ND-1000 spectrophotometer (OD 260 nm, NanoDrop Technologies, Wilmington, DE, USA) and standard denaturing agarose gel electrophoresis, respectively.

### lncRNA and mRNA microarrays and data analysis

Human lncRNAArraystar V4.0 was manufactured by Arraystar Inc. (MD, USA), and it covered more than 40,000 lncRNAs and more than 20,000 mRNAs in human genome. Transcript data were collected from authoritative sources, including NCBI RefSeq, Ensembl database, UCSC Genome Browser, and other sources from related literature. Every transcript was represented by 1–5 probesto improve confidence of statistical results. Microarray hybridization and collection of expression data were performed by KangChen Bio-tech, Shanghai, China.

Total RNAs from plasma were transcribed into complementary RNAs (cRNAs) and then labeled with Cyanine-3-CTP (Cy3) using the One-Color Quick Amp Labeling Kit (p/n 5190–0442, Agilent Technologies, Santa Clara, CA, USA) according to the manufacturer’s instruction. Labeled cRNAs were purified, and the concentration and specific activity were measured using a NanoDrop ND-1000 spectrophotometer and hybridized onto the microarray using the Agilent Gene Expression Hybridization Kit (p/n 5188–5242). The hybridized arrays were washed and scanned using the Agilent Microarray Scanner (p/n G2565BA). Raw data were collected from scanned images (TIF format) by Agilent Feature Extraction (version 11.0.1.1). After the quantile normalization of raw data by GeneSpringGX v12.0 (Agilent Technologies), lncRNAs and mRNAs with fold change (FC) cut-offs of ≥ 1.5 and significant *P* values of < 0.05, which were identified by paired *t* test, were selected for further analysis. Differentially expressed lncRNAs(DElncRNAs) and differentially expressed mRNAs (DEGs) derived from *t* test were identified through Volcano Plot filtering. Hierarchical clustering was performed by R package, including gplots and function heatmap 2.

### MiRNA sequencing and data analysis

MiRNA sequencing was carried out by KangChen Bio-tech using the Illumina Small RNA Sequencing Platform (San Diego, CA, USA) following the manufacturer’s instructions. Total RNAs from plasma were used to construct the miRNA sequencing library through the following steps: 3′ and 5′ adaptor ligation, cDNA synthesis followed by PCR amplification, and size selection (~ 135–155 bp PCR fragment, corresponding to ~ 15–35 nt small RNAs). Then single-stranded DNA denatured from the libraries were captured on Illumina flow cell, further amplified in situ as clusters, and sequenced for 51 cycles on an Illumina NextSeq 500 sequencer. The raw data were analyzed using routine algorithms (KangChen Biotech, Shanghai, China). Furthermore, differentially expressed miRNAs (DEmiRNAs) among groups were identified using edgeR (version 3.18.1) package with FC of ≥ 1.5 and *P* values of < 0.05.

### Construction of the ceRNA network

**Fig. 1:**
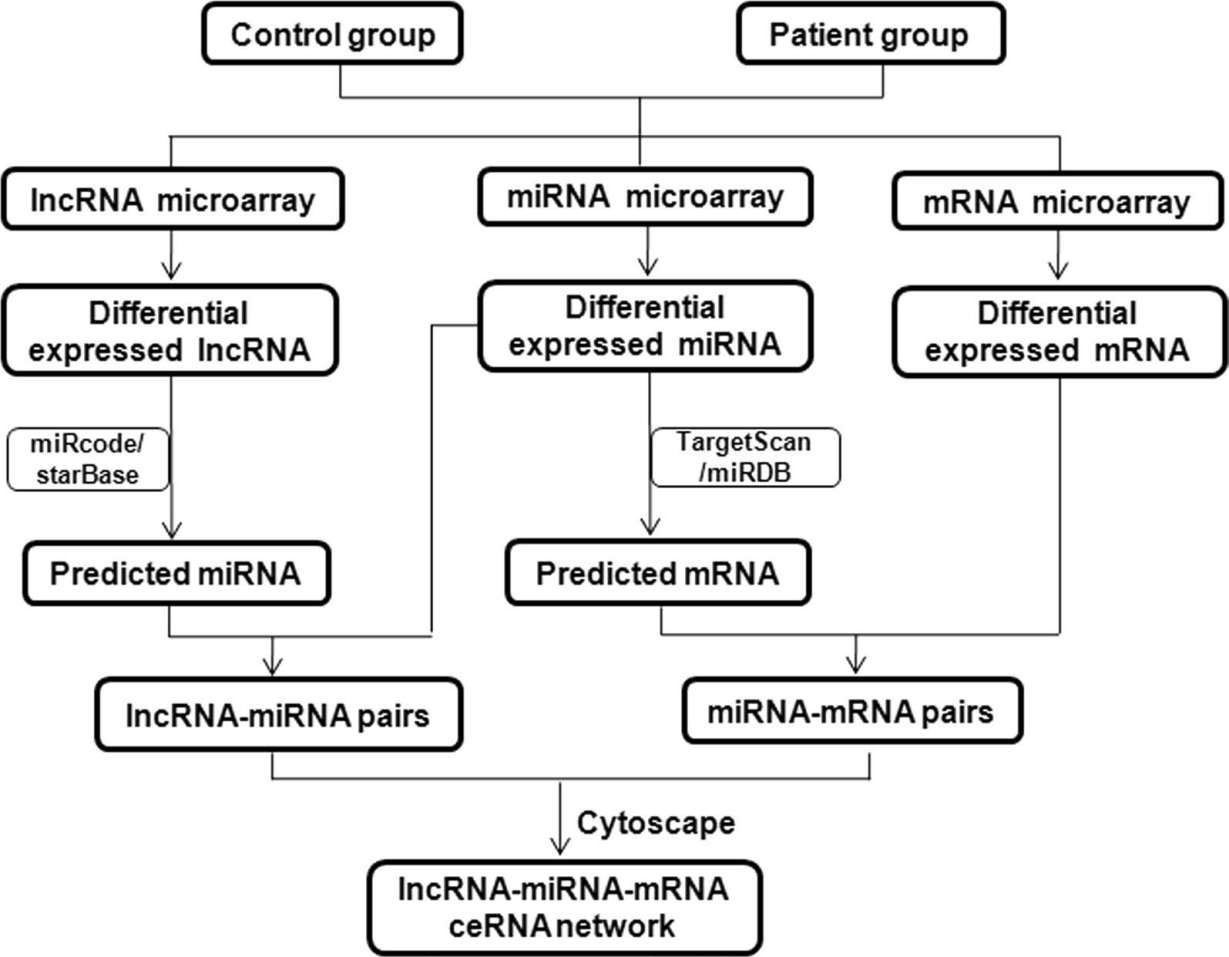
Flow chart of lncRNA–miRNA–mRNA ceRNA network analysis

The experimental procedure for microarray data acquisition, bioinformatic analysis, and network construction was performed following the flow chart shown in Fig. 1. In the ceRNA network, lncRNA and mRNA had the same expression trend. Thus, the correlation coefficient between DElncRNAs and DEGs was calculated using the Pearson correlation coefficient (PCC), and the pairs with PCC ≥ 0.9 and *P* < 0.05 were selected. The targeted miRNAsby DElncRNAs were searched using miRcode and StarBase, and only the DEmiRNA list was used to determine lncRNA–miRNA pairs. The targeted mRNAs by DEmiRNAs were searched using miRDB and TargetScan, and only the DEG list was used to determine miRNA–mRNA pairs. Then, theceRNA network was constructed, which showed that the lncRNA and mRNA were targeted, negatively co-expressed with the same miRNA among the selected lncRNA–mRNApairs, and visualized using Cytoscape V3.6.1. Then, all node degrees of the genes in ceRNA were calculated simultaneously using plugin CytoHubba.

### Gene Ontology (GO) and Kyoto Encyclopedia of Genes and Genomes (KEGG) pathway analysis

GO analysis and KEGG databases were carried out to analyze differentially expressed genes. GO project provides a vocabulary to describe gene functions (http://geneontology.org/), including three domains (biological process, cellular component, and molecular function). KEGG analysis allows genes to be mapped to pathways in metabolism, various cellular processes, and many human diseases. The Database for Annotation, Visualization and Integrated Discovery (DAVID) was used to find the potential function and underlying mechanism of differentially expressed genes [13, 14].

### Quantitative real-time polymerase chain reaction (qRT-PCR) validation

qRT-PCR was performed to validate hub genes. In brief, total RNA was reversetranscribed to cDNA using GoScript™ Reverse Transcription Mix (Promega), and then qRT-PCR was performed using GoTaq qPCR Master Mix (Promega) in the StepOne™ Real-Time PCR System (Thermo Scientific). The relative expression level of RNAs was normalized to the internal control β-actin, and all data were calculated using the 2^−ΔΔCt^ method. The primers for qRT-PCR were as follows: LINC00310 (GenBank Accession No. NR_027266) F: 5′-CAGCTTCAGAGAGTTCGAGTA-3′, R: 5′-CTCACAGAAACACCCAGAATA-3′; β-actin F: 5′-GTGGCCGAGGACTTTGATTG-3′, R: 5′-CCTGTAACAACGCATCTCATATT-3′.

### Statistical analyses

Data with a normal distribution were presented as mean ± SD. No normal data were compared with Mann–Whitney U test and expressed as median (P25–P75). Statistical analyses were carried out using SPSS v23.0 and R v3.6. *P* < 0.05 was considered significant.

## Results

### Identification of DElncRNAs, DEmiRNAs, and DEGs

**Fig. 2:**
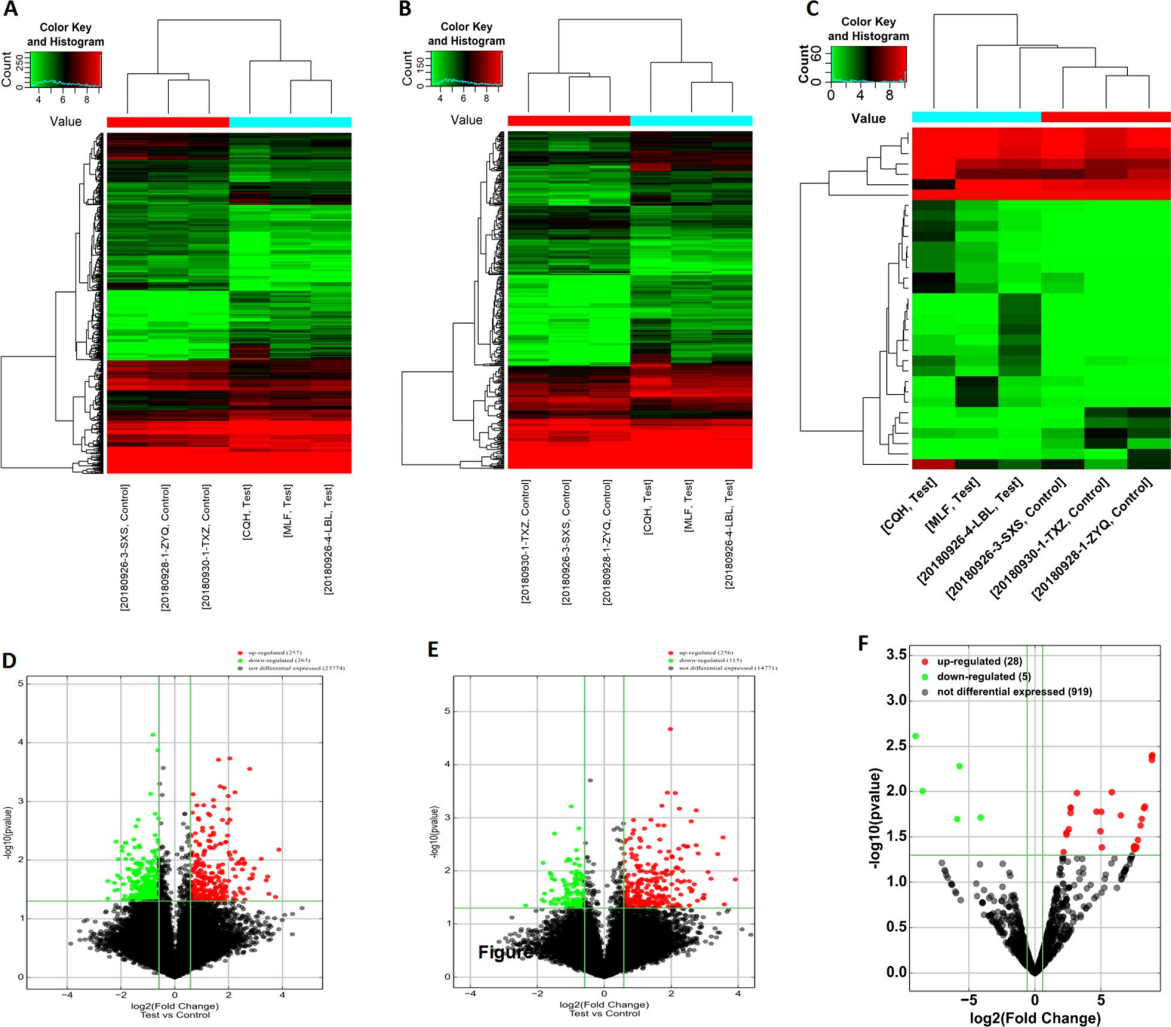
Cluster analysis and volcano plot of differently expressed RNAs. **a, d** lncRNAs; **b, e** mRNAs; **c, f** miRNAs. Upregulated genes are marked in light red; downregulated genes are marked in light green

In identifying the regulatory network of mRNA and ncRNA in HCM, the expression of lncRNAs and mRNAs was profiled by using microarray and miRNA and by sequencing three pairs of patients with HCM and healthy control plasma. After processing the raw data, a total of 520 lncRNAs were found to be significantly and differentially expressed betweenpatients with HCM and controls, of which 257 were upregulated and 263 were downregulated. A total of 371 DEGs were identified, with 256 upregulated and 115 downregulated. In addition, atotal of 33 DEmiRNAs were found, including 28 upregulated and five downregulated. The expression profiles of DElncRNAs, DEGs, and DEmiRNAs are depicted as heatmaps (Fig. 2a–c) and volcano dot plots (Fig. 2d–f) after normalization. The raw data of this study have been deposited in the GEO repository (GEO accession GSE197219), which are publicly accessible at https://www.ncbi.nlm.nih.gov/geo/query/acc.cgi?acc=GSE197219.

### Functional and pathway enrichment analyses of DEGs

**Fig. 3:**
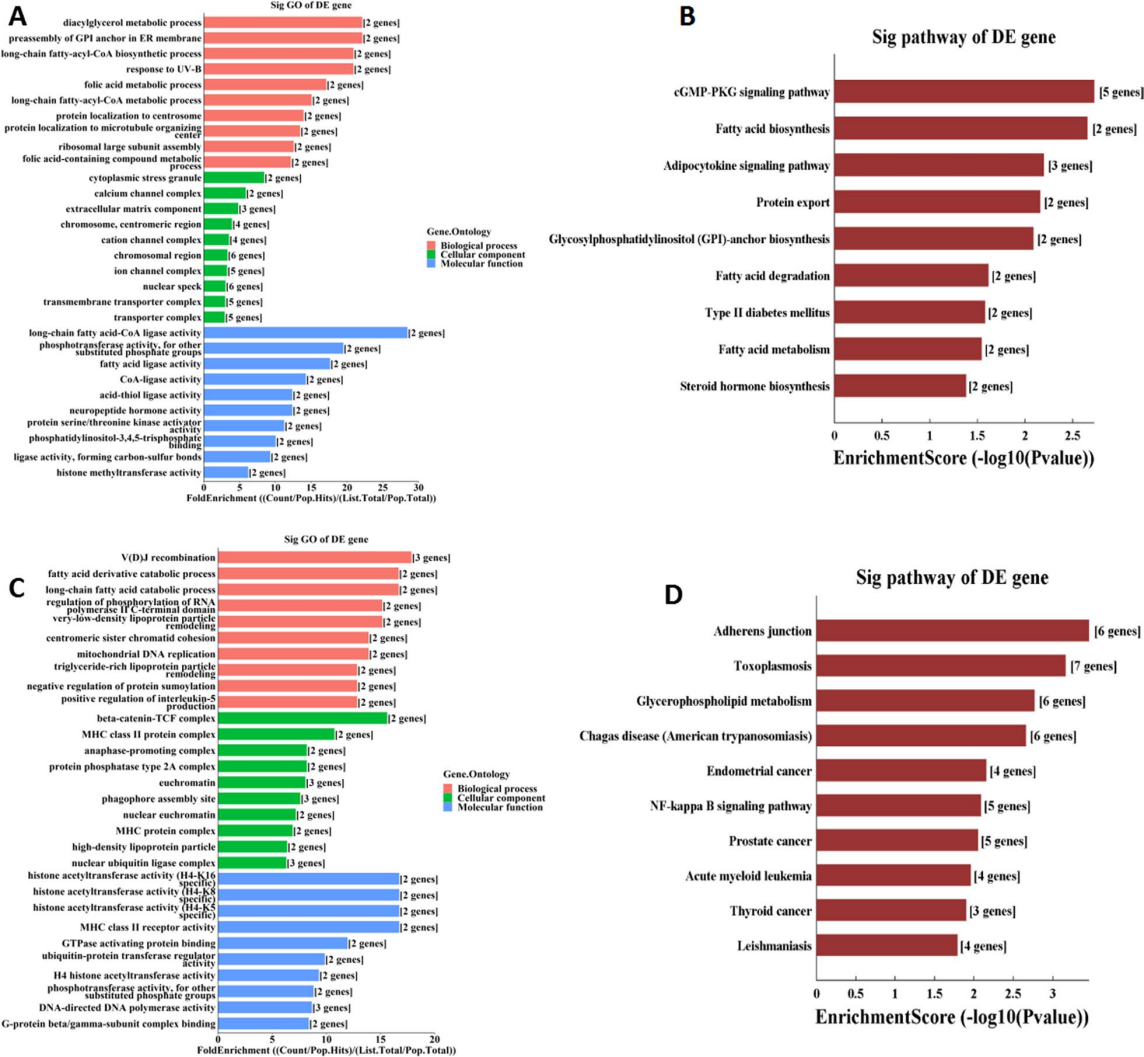
Top10 items of GO and KEGG enrichment analysis for differentially expressed genes in HCM. **a, b** Were GO and KEGG analyses of downregulated mRNAs. **c, d** Were GO and KEGG analyses of upregulated mRNAs

Enrichment analysis was applied to analyze plausible mechanisms and pathways to explore the potential function of DEGs. The top 10 items of GO analysis and KEGG analysis results are shown in Fig. 3. In brief, GO analysis revealed that downregulated mRNAs were enriched in biological processes (such as pre-assembly of the GPI anchor in the ER membrane, diacylglycerol metabolic process, and long-chain fatty-acyl-CoA biosynthesis), cellular component (including cytoplasmic stress granule, calcium channel complex, and extracellular matrix component), and molecular functions (including long-chain fatty acid-CoA ligase activity, phosphotransferase activity, other substituted phosphate groups, and fatty acid ligase activity; Fig. 3a). In addition, KEGG analysis was correlated with pathways, including cGMP-PKG signaling pathways, fatty acid biosynthesis, and adipocytokine signaling pathway (Fig. 3b). With regard to upregulated mRNAs, GO analysis presented that such mRNAs were primarily associated with biological processes (such as V(D)J recombination, cellular response to interleukin-4, and phosphatidylcholine biosynthesis), cellular component (such as euchromatin, catalytic complex, and nucleoplasm), and molecular functions (such as MHC class II receptor activity, histone acetyltransferase activity, and DNA-directed DNA polymerase activity; Fig. 3c). Moreover, KEGG analysis was enriched in several pathways, such as the adherens junction, glycerophospholipid metabolism, and NF-kappa B signaling pathway (Fig. 3d).

### Construction and dissection of the ceRNA network

**Fig. 4:**
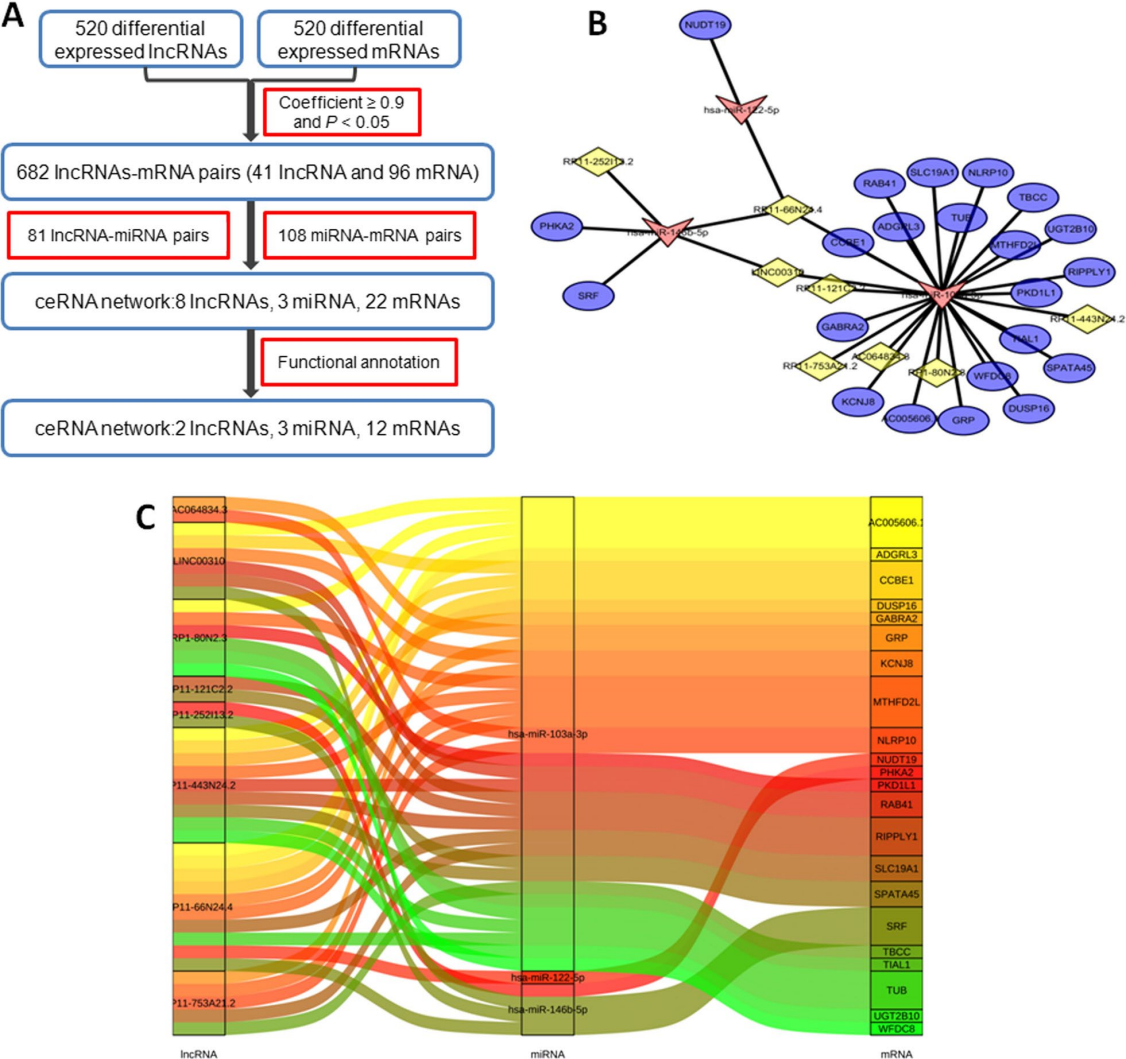
CeRNA network of lncRNA–miRNA–mRNA in HCM. **a** Flow chart for ceRNA network construction. **b** lncRNA–miRNA–ceRNA network visualized by Cytoscape. Yellow block represented lncRNA; red block represented miRNA, and purple block represented mRNA. **c** lncRNA–miRNA–ceRNA network visualized by Sankey diagram. HCM, hypertrophic cardiomyopathy; lncRNA, long noncoding RNA; miRNA, microRNA; mRNA, messenger RNA; ceRNA, competing endogenous RNA

Based on the results of bioinformatic analysis, the ceRNA network consists of DElncRNAs, DEmicroRNAs, and DEGs. First, a total of 682 lncRNA–mRNA pairs, including 41 lncRNA and 96 mRNA, were selected with PCC of ≥ 0.9 and *P* of < 0.05. Then, 81 lncRNA–miRNA pairs (including 46 DELncRNAs and seven DEmiRNAs) and 108 miRNA–mRNAs pairs (including six DEmiRNAs and 99 DEGs) were retrieved. Combining these pairs, the ceRNA network with eight lncRNAs, threemiRNAs, and 22 mRNAs was constructed on the basis of the established theory. Cytoscape and Sankey diagrams were used to visualize complex molecular interaction networks, and both diagrams clearly showed the potential interplay between eight lncRNAs and three miRNAs and between three miRNAs and 22 mRNAs (Fig. 4).

### Key lncRNA–miRNA–mRNA subnetwork

**Fig. 5:**
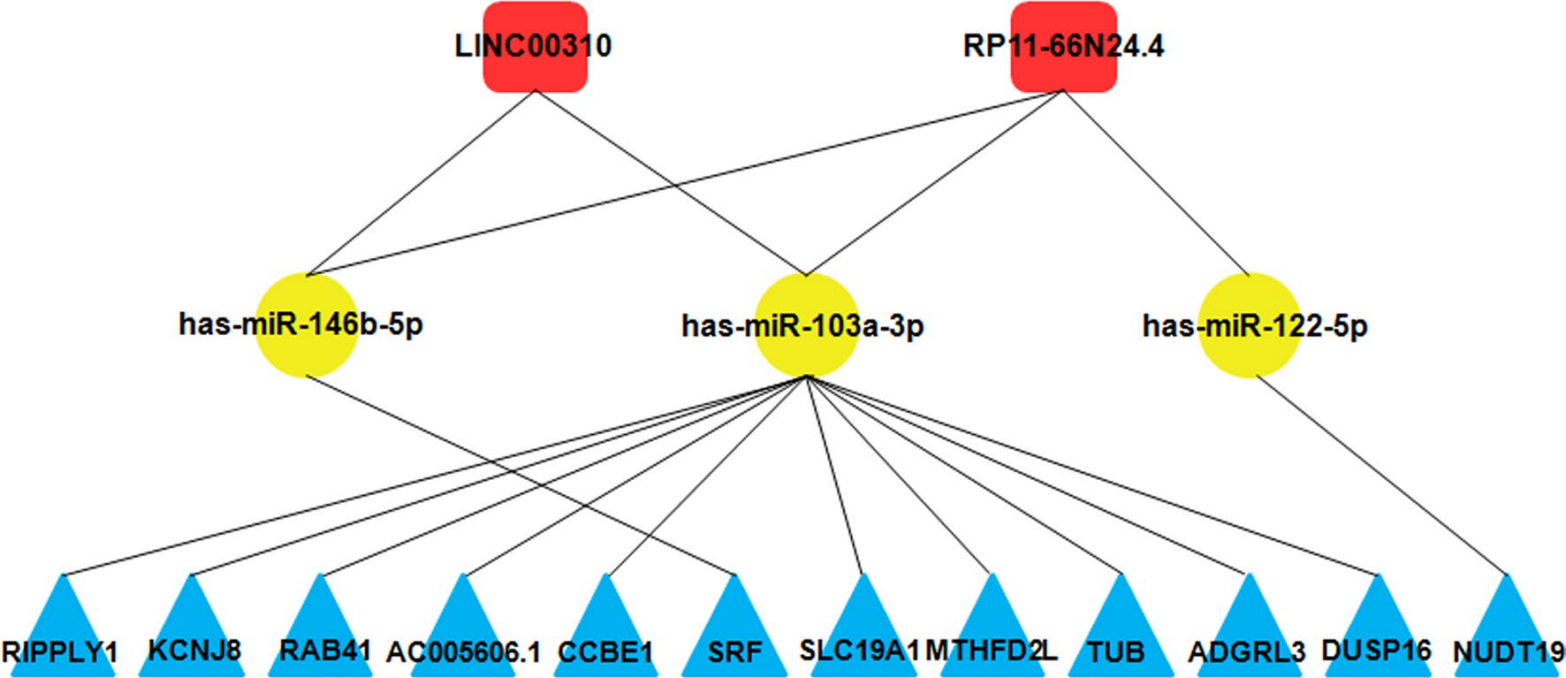
ceRNA subnetwork of lncRNA–miRNA–mRNA constructed from hub genes. Square block represented lncRNA; circular block represented miRNA, and triangle block represented mRNA. This ceRNA subnetwork included two lncRNA, three miRNA, and 12 mRNA

**Fig. 6:**
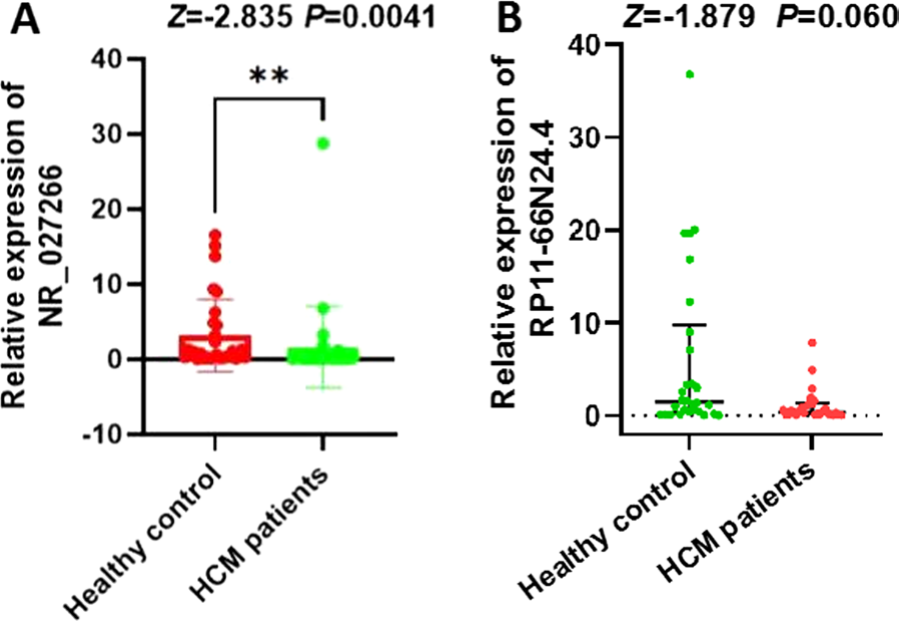
qRT-PCR analysis of LINC00310 and RP11-66N24.4between healthy controls and patients with HCM. **a** The LINC00310 level in patients with HCM was significantly downregulatedcompared with that in healthy controls. **b** No significant difference in RP11-66N24.4 was observed

Then, the degrees of each node in the ceRNA network was calculated to identify hub genes and their related networks, and the top 10% nodes were ranked on the basis of their degrees (Table 1). lncRNA RP11-66N24.4 and LINC00310 are among the ranked nodes, indicating that they may be the key lncRNAs in the ceRNA network, and the associated ceRNA may be the core of the whole ceRNA network. Then the subnetwork of hub genes was extracted out of the ceRNA network, which was composed of two lncRNA nodes, three miRNAnodes, and 12 mRNA nodes (Fig. 5, Table 2). qRT-PCR was performed usingplasma samples from 30 patients with HCM and 30 healthy control subjects to validate target lncRNAs. The results indicated that LINC00310 was significantly decreased in patients with HCM (*P* < 0.05, Fig. 6a). By contrast, no significant difference in RP11-66N24.4 was observed (*P* > 0.05, Fig. 6b). Then, GO and KEGG pathway annotations for LINC00310 were performed. For LINC00310, GO analysis revealed that biological processes were enriched in cardiovascular system development, sprouting angiogenesis, and circulatory system development (Additional file 1: Table S1). KEGG analysis demonstrated that LINC00310 might be associated with the cGMP-PKG signaling pathway [15] (*P* < 0.05, Additional file 2: Table S2).

**Table 1:** Top 10% genes of the ceRNA network in degree

Number	Gene type	Gene symbol	Degree
1	miRNA	hsa-miR-103a-3p	26
2	miRNA	hsa-miR-146b-5p	5
3	lncRNA	RP11-66N24.4	3
4	lncRNA	LINC00310	2
5	miRNA	hsa-miR-122-5p	2

**Table 2:** Reconstruction of lncRNA-associated ceRNA networks

lncRNA	miRNA	mRNA
LINC00310	hsa-miR-146b-5p	SRF
	hsa-miR-103a-3p	RIPPLY1
		KCNJ8
		RAB41
		AC005606.1
		CCBE1
RP11-66N24.4	hsa-miR-146b-5p	SRF
	hsa-miR-122-5p	NUDT19
	hsa-miR-103a-3p	SLC19A1
		MTHFD2L
		AC005606.1
		KCNJ8
		TUB
		ADGRL3
		CCBE1
		DUSP16

## Discussion

Once considered as transcriptional noise, lncRNAs are considered in gene regulation and cellular processes [16]. In the ceRNA network, miRNAs can affect lncRNA half-life by promoting their degradation, or lncRNAs can act as miRNA "sponges," reducing miRNA regulatory effect on target mRNAs [17]. lncRNA interplay with miRNAs is thoroughly studied in several physiological and disease states, whereas the relationship between ceRNA and HCM remains unclear.

Recently, many roles of lncRNAs on HCM are reported. For example, Chaer was downregulated, and it negatively regulated PRC2 function of H3K27me3 expression in failing mouse heart induced by pressure overload [18]. IGFBP5 might participate in the synthesis of myosin complex, kinesin binding, motor activity, and function via the regulation of actin cytoskeleton to regulate HCM progression [19]. A total of twolncRNAs (ENST00000458178.1 and ENST00000567093.1) primarily targeted miR-10a-5p, miR-30c-3p, miR-1247-5p, and miR-1268a, which were highly related to the development of HCM [20]. Although the roles of lncRNAs are important in the pathology of HCM, many lncRNAs should be studied under the circumstance of cardiac hypertrophy and remodeling.

To date, the possible role of specific lncRNAs was determined by several animal experiments and bioinformatic analyses in the production of HCM because of the difficulty in obtaining heart tissues [21]. In this study, plasma lncRNA and mRNA expression profiles of patients with HCM and healthy controls were obtained by using a microarray and by sequencingmiRNA. In identifying the regulatory effect of the lncRNA-associated ceRNA network in HCM, the predicted data from bioinformatic analysis overlapped the experimental data derived from plasma, andthe ceRNA network was constructed on the basis of the ceRNA theory. This strategy ensured the possibility and reliability of ceRNA regulatory mechanisms participating in HCM. The result showed that the lncRNA-associated ceRNA network consisted ofeight lncRNA nodes, three miRNA nodes, and 22 mRNAs nodes. Furthermore, the top 10% nodes were used to analyze the subnetwork where the hub lncRNA participates in more ceRNA interactions and plays a vital role in network organization structure. Therefore, the subnetwork of hub genes was composed of two lncRNA nodes, three miRNA nodes, and 12 mRNA nodes. Among these hub genes, LINC00310 was significantly decreased in patients with HCM by using qRT-PCR, and the potential targets included miR-103a-3p and miR-146b-5p in the ceRNA network.

The role of LINC00310 in HCM has not been previously reported. LINC00310 gene locus isobserved at the 21q22.11, and it has beenidentified as a top candidate gene associated with spontaneous coronary artery dissection [22], but the function and clinical significance of LINC00310 remain unknown. In this study, GO analysis showed that LINC00310 in the ceRNA network was associated with cardiovascular system development, sprouting angiogenesis, and circulatory system development, which suggested thatLINC00310 may be involved in cardiovascular diseases. The cardiovascular system is the first system that is developed in the growing embryo. The cardiovascular development involves a series of complicated processes to ensure rational formation of structures and timely changes in spatial form [23]. Any alterations in this process can cause congenital cardiovascular diseases (CVDs). In the ceRNA network, miR-103a-3p is elevated in the urine of patients with diabetes mellitus and upregulated in the plasma of patients with hypertension [24, 25], and modulated SNRK/NF-κB/p65 signaling promotingangiotensin II–induced renal inflammation and fibrosis [26]. Further study revealed that the downregulation of miR-146b-5p reduced the phenotypes of cardiac fibrosis in the MI mouse model [27]. However, the effect of these miRNAs on HCM is poorly understood, which needs further investigation.

The results on KEGG analysis showed that downregulated DEGs of the subnetwork and LINC00310 were enriched in the cGMP-PKG signaling pathway. cGMP-dependent PKI (PKG) is the primary cGMP mediator in the cardiovascular system, which functions by phosphorylating target proteins that play a significant role in regulating Ca^2+^ signaling, relaxation, and contraction of cardiac myocytes and vascular smooth muscle cells;inhibiting inflammation; and reducing oxidative stress [28, 29]. However, no data regarding the association between the cGMP-PKG signaling pathway and HCM are available, and further studies are necessary. As for mRNAs shown in the results of KEGG analysis of LINC00310, accumulated data have shown that KCNJ8 and SRF participate in the progression of pathologic cardiac hypertrophy. Previous studies have demonstrated that Cantu syndrome–associated mutations in KCNJ8 resulted in cardiac hypertrophy, but the pathogenesis remains unclear [30]. SRF can trigger the atrialnatriuretic factor, α-MHC, Acta 1, BNP, and β-MHC in isolated cardiomyocytes, which suggested that SRF is crucial to the regulation and induction of genes associated with the pathogenesis of pathologic cardiac hypertrophy [31]. Transgenic mice with moderate cardiac-specific overexpression of the human SRF gene manifested significant cardiac hypertrophy and premature death [32]. These results indicated that the LINC00310-miR-103a/146b-5p axis may play a significant role in the development of HCM, which is the first report of its role in the ceRNA network of HCM.

Although the LINC00310-associated ceRNA network was identified, which may be involved in the pathogenesis of HCM in this study, in vitro and in vivo effects should be further demonstrated experimentally to elucidate the exact mechanism of ceRNA networks in HCM, that is, both lncRNAs bind miRNAs by performing the binding assay using luciferase with a mutated sequence of the miRNA-binding site.

In this study, the lncRNA-associated ceRNA network was constructed, and the LINC00310-miR-103a/146b-5p axis in the network may be a crucial RNA transcript in HCM pathogenesis. This study could unravel the pathogenesis of HCMand provide potential target genes of HCM pathogenesis.

## Supplementary Information

**Additional file 1: Table S1**. The results of GO analysis for LINC00310.

**Additional file 2: Table S2**. The results of KEGG analysis for LINC00310.

## Data Availability

The raw data supporting the conclusions of this article have been deposited in the GEO repository (GEO accession GSE197219), which are publicly accessible at https://www.ncbi.nlm.nih.gov/geo/query/acc.cgi?acc=GSE197219, enter uzwfeeqifvsphmr token into the box.

## Abbreviations

HCM: Hypertrophic cardiomyopathy
lncRNA: Long noncoding RNA
ceRNA: Competing endogenous RNA
miRNA: MicroRNA
DElncRNAs: Different expression profiles of lncRNAs
DEGs: Different expression profiles of messenger RNAs (mRNA)
DEmiRNAs: Different expression profiles of microRNAs
qPCR: Real-time quantitative PCR detecting system
GO: Gene Ontology
KEGG: Kyoto Encyclopedia of Genes and Genomes
LV: Left ventricle hypertrophy
FC: Fold change
PCC: Pearson correlation coefficient
